# Cardiometabolic risk factors among HIV patients on antiretroviral therapy

**DOI:** 10.1186/1476-511X-12-50

**Published:** 2013-04-10

**Authors:** James N Kiage, Douglas C Heimburger, Christopher K Nyirenda, Melissa F Wellons, Shashwatee Bagchi, Benjamin H Chi, John R Koethe, Donna K Arnett, Edmond K Kabagambe

**Affiliations:** 1 Department of Medicine, Division of Epidemiology, Vanderbilt University, Nashville, TN 37203, USA; 2 Department of Medicine, Division of Diabetes, Endocrinology and Metabolism, Vanderbilt University, Nashville, TN 37203, USA; 3 Department of Medicine, Division of Infectious Diseases, Vanderbilt University, Nashville, TN 37203, USA; 4 Institute for Global Health, Vanderbilt University, Nashville, TN 37203, USA; 5 Department of Nutrition Sciences, University of Alabama at Birmingham, Birmingham, AL 35294, USA; 6 Ndola Central Hospital and Copperbelt University School of Medicine, Ndola, Zambia; 7 Department of Medicine, University of Maryland, Baltimore, MD 21201, USA; 8 Centre for Infectious Disease Research in Zambia, Lusaka, Zambia; 9 Department of Obstetrics and Gynecology, University of North Carolina at Chapel Hill, Chapel Hill, NC 27599, USA; 10 Department of Epidemiology, University of Alabama at Birmingham, Birmingham, AL 35294, USA

**Keywords:** Lipids, cART, Cardiometabolic risk, Zambia

## Abstract

**Background:**

HIV and combination antiretroviral therapy (cART) may increase cardiovascular disease (CVD) risk. We assessed the early effects of cART on CVD risk markers in a population with presumed low CVD risk.

**Methods:**

Adult patients (n=118) in Lusaka, Zambia were recruited at the time of initiation of cART for HIV/AIDS. Cardiometabolic risk factors were measured before and 90 days after starting cART. Participants were grouped according to cART regimens: Zidovudine + Lamivudine + Nevirapine (n=58); Stavudine + Lamivudine + Nevirapine (n=43); and ‘other’ (Zidovudine + Lamivudine + Efavirenz, Stavudine + Lamivudine + Efavirenz, Tenofovir + Emtricitabine + Efavirenz or Tenofovir + Emtricitabine + Nevirapine, n=17). ANOVA was used to test whether changes in cardiometabolic risk markers varied by cART regimen.

**Results:**

From baseline to 90 days after initiation of cART, the prevalence of low levels of high-density lipoprotein cholesterol (<1.04 mmol/L for men and <1.30 mmol/L for women) significantly decreased (78.8% vs. 34.8%, *P*<0.001) while elevated total cholesterol (TC ≥5.18 mmol/L, 5.1% vs. 11.9%, *P*=0.03) and the homeostasis model assessment of insulin resistance ≥3.0 (1.7% vs. 17.0%, *P*<0.001) significantly increased. The prevalence of TC:HDL-c ratio ≥5.0 significantly decreased (44.9% vs. 6.8%, *P*<0.001). These changes in cardiometabolic risk markers were independent of the cART regimen.

**Conclusion:**

Our results suggest that short-term cART is associated with a cardioprotective lipid profile in Zambia and a tendency towards insulin resistance regardless of the cART regimen.

## Introduction

HIV/AIDS patients have increased risk for adverse cardiovascular outcomes and diabetes [1-3]. Both the HIV infection and combination antiretroviral therapy (cART) are independently associated with increased cardiometabolic risk [4,5].

Studies, mainly from developed countries, have shown that certain cART regimens, particularly combinations containing protease inhibitors, are associated with increased serum triglycerides (TG), low-density lipoprotein cholesterol (LDL-c) and total cholesterol (TC) as well as insulin resistance, while little or no effect is seen on high-density lipoprotein cholesterol (HDL-c) [6-8]. This constellation of dyslipidemia, especially elevation of TC:HDL-c ratio, and insulin resistance is thought to enhance the process of atherosclerosis, thus partly explaining the link between cART and adverse cardiometabolic outcomes [9-11]. Although protease inhibitor sparing combinations have also been associated with similar lipid changes, they result in substantial HDL-c elevation and relatively lower elevations of TG and LDL-c [6]. This lipid profile, especially the elevation of HDL-c, is thought to be beneficial to cardiovascular health [6,11,12].

However, few studies have explored the association between cART and cardiovascular health within resource-constrained settings where the majority of HIV/AIDS cases reside and where the prevalence of traditional cardiovascular risk factors, such as excess adiposity, a high fat diet, and smoking, may be lower [13-15]. Findings from developed countries may not hold in resource-poor settings due to factors that could potentially modify cardiovascular risk e.g., age of the population, differences in HIV subtypes, diet, lifestyle as well as genetics [14]. The ongoing expansion of the cART programs within resource-limited settings highlights the need to quantify cardiometabolic risk associated with different antiretroviral combinations in these settings [15,16].

Using serum lipids and markers of insulin resistance as cardiometabolic risk markers, we investigated changes in cardiometabolic risk following cART among treatment-naïve HIV positive patients initiating therapy in Zambia. We also tested whether the changes in cardiometabolic risk markers varied by cART regimen.

## Methods

### Ethics statement

The study was approved by the Institutional Review Board of the University of Alabama at Birmingham (UAB) and the Research Ethics Committee of the University of Zambia. All participants gave written informed consent.

### Study design and population

The current analysis is based on data from the Diet, Genetic Polymorphisms in Lipid-Metabolizing Enzyme genes, and Antiretroviral Therapy-Related Dyslipidemia (DGPLEAD) study which was carried out in Chawama Clinic, Lusaka, Zambia. The study has been described elsewhere [17]. In brief, 210 cART-naïve HIV/AIDS patients were recruited between January and December 2007. Men and women aged 16.5-60 years who were eligible to begin cART according to the Zambia HIV national guidelines and had BMI ≥16 kg/m^2^ and CD4+ lymphocyte count ≥50 cells/μL were invited into the study. All participants who gave consent were enrolled. The aforementioned BMI and CD4+ criteria were used because another study in the same clinic simultaneously recruited participants with BMI and CD4+ levels below these thresholds [18]. cART was prescribed according to the Zambian national guidelines at the time. Regimens comprised 2 nucleoside reverse transcriptase inhibitors (NRTI), for example zidovudine [AZT] and lamivudine [3TC] or stavudine [D4T] and lamivudine [3TC], and a non-nucleoside reverse transcriptase inhibitor, for example efavirenz [EFV] or nevirapine [NVP]. Tenofovir [TDF] and emtricitabine [FTC] were introduced into the first-line NRTI backbone in July 2007, while the study was underway [19]. No protease inhibitors were included in the regimens.

### Data collection

At the initial visit, data on smoking, physical activity and alcohol intake were collected using standardized questionnaires. In addition, dietary intake was assessed using 24-hour dietary recalls; the Nutrition Data System for Research software was used to determine dietary nutrient content. Zambian foods not in the database were substituted with similar foods in the database using a recommended guideline [17]. Also added into the database were recipes for common foods which were collated through focus group meetings.

Anthropometric measurements including weight, height, waist circumference, hip circumference and mid-upper arm circumference were recorded as an average of two measurements made by a trained staff member. A blood specimen was taken from each participant after an 8-hour fast to determine lipid and metabolic profiles. Participants were followed, and their lipid and metabolic profiles and vital status were reassessed at 90 days following cART initiation.

### Laboratory tests

Lipids and glucose were assayed using the Roche Cobas Integra 400+ auto analyzer (Roche Diagnostics, Indianapolis, IN, USA). An enzymatic colorimetric assay was used to measure TG, LDL-c and TC concentrations while a homogeneous enzymatic colorimetric assay was used to measure HDL-c. In addition, the hexokinase enzyme method was used to measure glucose concentration while insulin was assayed using chemiluminescence on the Roche Elecsys 2010 autoanalyzer. Serum albumin, creatinine and high-sensitivity C-reactive protein (CRP) were measured on a Roche Modular P analyzer using bromocresol purple assay for albumin and immunoturbidimetric assay for CRP (Roche Diagnostics, Indianapolis, IN, USA).

### Statistical analysis

Only participants with both baseline and 90-day lipid and other metabolic measurements were included in the current analysis. cART regimens were grouped into 3 categories as follows: AZT+3TC+NVP, D4T+3TC+NVP and ‘other’ (AZT+3TC+EFV, D4T+3TC+EFV, TDF+FTC+EFV or TDF+FTC+NVP). We compared baseline characteristics of participants by cART regimen using one-way ANOVA and Kruskal-Wallis tests for continuous variables and chi-square tests for categorical variables. We then tested lipid, insulin and glucose changes from baseline to 90 days using paired t-tests and Wilcoxon signed-rank tests. We stratified the data by the antiretroviral combinations and again tested whether changes were significant using Wilcoxon signed-rank tests. We used ANOVA models with robust variance estimators to determine whether the magnitude of changes in cardiometabolic markers varied by cART regimen; change from baseline to 90-day was the dependent variable and cART regimen and sex were the independent variables. Sex was included in these models because of the significant differences in sex by cART regimen (Table 1).

**Table 1:** Baseline characteristics of the DGPLEAD study overall and by combination antiretroviral therapy

		**Combination antiretroviral therapy**			
**Variable**	**Overall (n = 118)**	**AZT+3TC+NVP (n = 58)**	**D4T+3TC+NVP (n = 43)**	**Other ‡ (n = 17)**	P *
Age, years†	35.0 ± 7.9	36.7 ± 7.5	33.3 ± 7.3	33.5 ± 9.7	0.07
Gender, % female	55.9	41.4	72.1	64.7	0.01
BMI, kg/m^2^	20.2 ± 2.7	20.2 ± 2.7	20.0 ± 2.4	20.3 ± 3.5	0.86
BMI, %					
< 18.5 kg/m^2^	29.7	27.6	27.9	41.2	0.63
18.5 - 24.9 kg/m^2^	63.6	67.2	65.1	47.1	
≥ 25.0 kg/m^2^	6.8	5.2	7.0	11.8	
Weight, kg	54.9 ± 8.7	57.2 ± 9.0	51.9 ± 7.1	54.4 ± 9.7	0.01
Height, m	1.65 ± 0.08	1.68 ± 0.07	1.61 ± 0.07	1.64 ± 0.07	< 0.001
Waist circumference, cm	72.9 ± 6.7	73.8 ±7.2	72.7 ± 6.0	70.2 ± 6.3	0.16
Hip circumference, cm	89.1 ± 7.0	90.1 ± 7.4	88.1 ± 6.2	88.2 ± 7.7	0.32
MUAC, cm	24.9 ± 2.6	25.4 ± 2.7	24.2 ± 2.1	24.9 ± 2.9	0.06
Current smoker, % †	4.4	7.1	2.3	0.0	0.33
Current drinker, % †	9.6	14.3	4.7	6.3	0.24
Total energy intake, kcal/day	1750 ± 651	1840 ± 641	1620 ± 604	1770 ± 773	0.25
Total fat, % energy/day	31.7 ± 10.8	29.3 ± 9.5	34.0 ± 11.2	34.5 ± 12.4	0.05
MUFA, % energy/day	9.1 ± 3.5	8.3 ± 3.0	9.8 ± 4.2	9.8 ± 3.1	0.07
PUFA, % energy/day	14.1 ± 5.9	12.8 ± 5.0	15.2 ± 5.9	15.4 ± 7.9	0.07
Saturated fat, % energy/day	6.3 ± 2.5	6.0 ± 2.5	6.6 ± 2.7	6.8 ± 2.4	0.37
Carbohydrates, % energy/day	56.1 ± 12.4	58.0 ± 11.5	54.2 ± 12.8	54.5 ± 14.3	0.27
Proteins, % energy/day	12.9 ± 3.9	13.4 ± 4.2	12.6 ± 4.0	12.0 ± 2.2	0.34
CD4 count, cells/μL	136 ± 50	137 ± 45	133 ± 51	138 ± 65	0.87
CRP, mg/L †	9.48 [2.04, 25.94]	5.62 [1.31, 17.36]	16.62 [5.51, 44.31]	4.05 [1.64, 25.62]	0.01
CRP ≥ 3.0 mg/L, % †	70.4	63.5	84.6	58.8	0.05
Albumin, g/dL †	3.11 ± 0.71	3.44 ± 0.59	2.67 ± 0.64	3.14 ± 0.67	< 0.001
Albumin < 3.5 g/dL, % †	65.7	48.1	89.7	64.7	< 0.001
Creatinine ≥ 2.0 mg/dL, % †	0.93	0.0	2.6	0.0	0.41
Fasting insulin, μU/mL †	3.00 [1.90, 5.40]	3.00 [2.00, 5.00]	3.00 [1.50, 5.00]	2.60 [1.20, 5.90]	0.80
Fasting glucose, mmol/L †	3.80 [3.40, 4.10]	3.90 [3.60, 4.30]	3.70 [3.40, 4.10]	3.60 [3.00, 3.90]	0.02
HOMA-IR	0.51 [0.30, 0.98]	0.53 [0.33, 0.93]	0.50 [0.28, 1.00]	0.45 [0.16, 0.98]	0.63
Total cholesterol, mmol/L	3.56 ± 0.83	3.68 ± 0.75	3.42 ± 0.92	3.52 ± 0.86	0.27
Triglycerides, mmol/L	1.02 [0.85, 1.37]	1.01 [0.86, 1.33]	1.16 [0.88, 1.60]	0.95 [0.77, 1.05]	0.15
LDL-cholesterol, mmol/L	2.12 ± 0.75	2.26 ± 0.62	1.93 ± 0.89	2.10 ± 0.68	0.10
HDL-cholesterol, mmol/L	0.72 [0.52, 1.09]	0.80 [0.61, 1.15]	0.65 [0.34, 0.92]	0.76 [0.57, 1.03]	0.08
TC:HDL-c ratio	4.77 [3.51, 6.30]	4.54 [3.44, 5.84]	5.32 [3.97, 8.93]	4.34 [3.33, 5.82]	0.15

DGPLEAD, Diet, Genetic Polymorphisms in Lipid-Metabolizing Enzyme genes, and Antiretroviral Therapy-Related Dyslipidemia; BMI, Body Mass Index; MUAC, Mid Upper Arm Circumference; MUFA, Monounsaturated Fatty Acids; PUFA, Polyunsaturated Fatty Acids; CRP, C-reactive Protein; LDL, Low Density Lipoprotein; HDL, High Density Lipoprotein; TC, Total Cholesterol; HOMA-IR, Homeostatic model assessment of insulin resistance.

Values are reported as mean ± standard deviation, median [25^th^ percentile, 75^th^ percentile] or percent.

‡ AZT + 3TC + EFV, D4T + 3TC + EFV, TDF + FTC + EFV, or TDF+FTC+NVP.

* *P*-values for the comparison between cART regimens were obtained through one-way ANOVA or Kruskal-Wallis tests for continuous variables and from Chi-square tests for categorical variables.

† Variables with missing values – 1 for glucose, 2 for age, 3 for both smoking and alcohol status, 10 for CRP, albumin and creatinine and 20 for insulin.

We used the US National Cholesterol Education Program Adult Treatment Panel III guidelines [10] to define abnormal lipid values as follows: TG ≥1.70 mmol/L (150 mg/dL), LDL-c ≥3.37 mmol/L (130 mg/dL), TC ≥5.18 mmol/L (200 mg/dL), and HDL-c <1.04 mmol/L (40 mg/dL) in men and <1.30 mmol/L (50 mg/dL) in women. We also defined TC:HDL-c ratio ≥5.0 and glucose ≥5.55 mmol/L (100 mg/dL) as abnormal [13,20]. Participants with a homeostasis model assessment of insulin resistance (HOMA-IR) index ≥3.0 were considered to be insulin resistant [21,22]. HOMA-IR was calculated using the following formula: fasting serum insulin (μU/mL) × fasting plasma glucose (mmol/L)/22.5 [23]. We used McNemar’s test to compare the prevalence of the abnormal values between baseline and 90 days. We repeated the analysis for HDL-c after redefining low HDL-c as <1.04 mmol/L (<40 mg/dL) for both men and women to enable comparison with previous studies.

To determine the potential for selection bias, we compared baseline characteristics of participants who were included to those who were excluded from the current analysis. All analyses were done using SAS version 9.2 (SAS Institute Inc., Cary, NC, USA); differences were considered significant at *P*≤0.05.

## Results

Of the 210 participants recruited into the DGPLEAD study, 134 had both baseline and 90-day information. We excluded 16 more participants for missing data on TC, HDL-c, LDL-c and medications, leaving 118 participants for the current analysis. Their summary statistics are shown in Table 1.

### Baseline characteristics

The mean age±SD of the study population was 35.0±7.9 years; 55.5% were women. The majority of the participants (85.6%) were either on AZT+3TC+NVP or D4T+3TC+NVP. Participants in various antiretroviral combination groups were similar (*P*>0.05) with regard to age, body mass index (BMI; weight (kg)/height[m]^2^), waist circumference, hip circumference, mid-upper arm circumference, smoking, alcohol drinking, nutrient intake and lipid profiles at baseline. The majority of participants on AZT+3TC+NVP were men while women were the majority in the D4T+3TC+NVP and ‘other’ cART regimens. Participants on D4T+3TC+NVP, had a lower mean weight (*P*=0.01) and height (*P*<0.001), had a higher median CRP (*P*=0.01) and a higher proportion of high CRP (≥3.0 mg/dL; *P*=0.05) compared to the participants on AZT+3TC+NVP or ‘other’ cART regimens. In addition, participants on D4T+3TC+NVP had a lower mean serum albumin (*P*<0.001) and a higher proportion of low serum albumin (<3.5 g/dL; *P*<0.001).

### Changes from baseline to 90 days

Table 2 shows lipid and other metabolic changes from baseline to 90 days. Total cholesterol, LDL-c and HDL-c concentrations significantly increased (*P*<0.001, *P*=0.02 and *P*<0.001, respectively); the increase in HDL-c was proportionately greater than that of TC, yielding a significant decrease in the TC:HDL-c ratio (*P*<0.001). Insulin and HOMA-IR doubled (*P*<0.001 in both cases), while glucose and BMI increased significantly but less substantially (*P*=0.05 and *P*<0.001, respectively). There was no significant change in TG concentrations (*P*=0.36).

**Table 2:** Lipid and metabolic changes from baseline to 90 days of combination antiretroviral therapy

**Variable**	**Baseline (n = 118)**	**End (n = 118)**	P* for change
HDL-cholesterol, mmol/L	0.72 [0.52, 1.09]	1.34 [1.09, 1.70]	< 0.001
LDL-cholesterol, mmol/L	2.12 ± 0.75	2.28 ± 0.59	0.02
Total cholesterol, mmol/L	3.56 ± 0.83	4.09 ± 0.86	< 0.001
Triglycerides, mmol/L	1.02 [0.85, 1.37]	1.03 [0.79, 1.37]	0.36
TC:HDL-c ratio	4.77 [3.51, 6.30]	2.93 [2.38, 3.69]	< 0.001
Insulin, μU/mL	3.00 [1.90, 5.40]	6.40 [3.40, 12.50]	< 0.001
Glucose, mmol/L	3.80 [3.40, 4.10]	4.00 [3.40, 4.50]	0.048
HOMA-IR	0.51 [0.30, 0.98]	1.12 [0.61, 2.21]	< 0.001
BMI, kg/m^2^	20.15 ± 2.70	20.89 ± 2.92	< 0.001
Weight, kg	54.9 ± 8.7	56.8 ± 9.0	< 0.001

Values are reported as mean ± standard deviation or median [25^th^ percentile, 75^th^ percentile].

* *P*-values obtained from paired t-tests or Wilcoxon Signed rank tests.

Analyses stratified by cART regimen (Table 3) showed significant increases (*P*<0.05) in HDL-c and decreases in TC:HDL-c ratio from baseline to 90 days in all cART regimens. In addition, there were increases in TC, insulin, HOMA-IR and BMI across all 3 cART regimens, though this increase was statistically significant (*P*<0.05) only in the AZT+3TC+NVP and D4T+3TC+NVP cART groups and not in the ‘other’ group (*P*>0.05). Furthermore, only the group on D4T+3TC+NVP had a significant increase in LDL-c (*P*=0.02), while none of the groups exhibited a significant change (*P*>0.05) in TG.

**Table 3:** Comparison of metabolic changes by period and combination antiretroviral therapy regimen in the DGPLEAD study

	**AZT+3TC+NVP (n = 58)**			**D4T+3TC+NVP (n = 43)**			**Other (n = 17)**			Global P**
**Variable**	**Baseline**	**End**	P*	**Baseline**	**End**	P*	**Baseline**	**End**	P*	
HDL-c, mmol/L	0.80 [0.61, 1.15]	1.42 [1.17, 1.73]	< 0.001	0.65 [0.34, 0.92]	1.27 [1.02, 1.70]	< 0.001	0.76 [0.57, 1.03]	1.22 [0.96, 1.47]	0.003	0.22
LDL-c, mmol/L	2.24 [1.76, 2.52]	2.25 [2.00, 2.75]	0.51	2.01 [1.44, 2.42]	2.27 [1.81, 2.61]	0.007	2.06 [1.71, 2.58]	2.14 [1.84, 2.18]	0.41	0.13
TC, mmol/L	3.59 [3.23, 3.99]	4.00 [3.50, 4.68]	< 0.001	3.46 [2.85, 4.08]	4.13 [3.59, 4.58]	< 0.001	3.40 [2.86, 3.87]	3.50 [3.40, 3.73]	0.77	**0.04**
TC:HDL-c ratio	4.54 [3.44, 5.84]	2.78 [2.34, 3.49]	< 0.001	5.32 [3.97, 8.93]	3.08 [2.85, 3.94]	< 0.001	4.34 [3.33, 5.82]	2.73 [2.31, 3.00]	0.001	0.36
TG, mmol/L	1.01 [0.86, 1.33]	1.06 [0.70, 1.41]	0.93	1.16 [1.88, 1.60]	1.05 [0.85, 1.44]	0.41	0.95 [0.77, 1.05]	0.91 [0.68, 1.14]	0.17	0.33
Insulin, μU/mL	3.00 [2.00, 5.00]	6.70 [4.00, 12.50]	< 0.001	3.00 [1.50, 5.00]	7.65 [3.00, 13.45]	< 0.001	2.60 [1.20, 5.90]	4.65 [3.15, 9.70]	0.07	0.18
Glucose, mmol/L	3.90 [3.60, 4.30]	4.20 [3.60, 4.60]	0.37	3.70 [3.40, 4.10]	3.80 [3.20, 4.60]	0.23	3.60 [3.00, 3.90]	3.70 [3.50, 4.20]	0.17	0.79
HOMA-IR	0.53 [0.33, 0.93]	1.12 [0.62, 2.41]	< 0.001	0.50 [0.28, 1.00]	1.39 [0.49, 2.49]	< 0.001	0.45 [0.16, 0.98]	0.72 [0.49, 1.53]	0.08	0.07
BMI, kg/m^2^	20.1 [18.2, 21.5]	20.3 [19.1, 22.2]	0.001	19.6 [18.4, 21.1]	20.6 [19.2, 22.4]	< 0.001	19.4 [17.6, 22.4]	20.1 [18.6, 22.3]	0.49	0.26
Weight, kg	55.3 [51.0, 62.0]	58.0 [53.5, 64.0]	0.001	52.0 [46.0, 55.0]	56.0 [47.0, 59.0]	< 0.001	53.0 [49.0, 60.5]	55.0 [50.0, 57.0]	0.49	0.30

Values are reported as median [25^th^ percentile, 75^th^ percentile].

* *P*-values obtained through Wilcoxon signed rank tests.

** *P*-values are from ANOVA with robust variance estimators comparing changes from baseline to 90 days after initiating cART and are adjusted for sex.

DGPLEAD, Diet, Genetic Polymorphisms in Lipid-Metabolizing Enzyme genes, and Antiretroviral Therapy-Related Dyslipidemia.

Except for TC (*P*=0.04), none of the changes in cardiometabolic risk markers varied significantly by treatment group in analyses adjusted for sex (Table 3). In models further adjusted for age and BMI, the *P*-values for the treatment regimen remained essentially unchanged.

### Prevalence of lipid and metabolic abnormalities at baseline and 90 days

**Figure 1:**
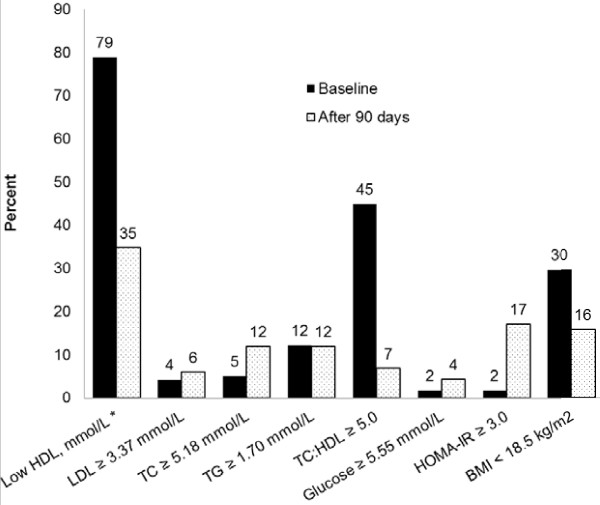
**Prevalence of abnormal lipid and other cardiometabolic risk factors at baseline and 90 days following cART initiation in the DGPLEAD study.** Comparing baseline measurements to those at 90 days following initiation of therapy, there was a significant change in the proportion of patients with low HDL-c (*P*<0.001), TC ≥5.18 mmol/L (*P*=0.03), TC:HDL-c ≥ 5.0 (*P*<0.001), HOMA-IR ≥3.0 (*P*<0.001) and BMI <18.5 (*P*<0.001). There was no significant change (*P*>0.05) in LDL-c ≥3.37 mmol/L, triglycerides ≥1.70 mmol/L and glucose ≥5.55 mmol/L. * Low HDL-c was defined as <1.04 mmol/L in men and <1.30 mmol/L in women. BMI, Body Mass Index; HDL-c, High Density Lipoprotein Cholesterol; HOMA-IR, Homeostasis Model Assessment of Insulin Resistance; LDL-c, Low Density Lipoprotein Cholesterol; TC, Total Cholesterol.

As shown in Figure 1, the prevalence of TC ≥5.18 mmol/L significantly increased (5.1% vs. 11.9%, *P*=0.02) while the prevalence of low HDL-c (<1.04 mmol/L (<40 mg/dL) in men and <1.30 mmol/L (<50 mg/dL) in women), TC:HDL-c ≥5.0 and BMI <18.5 kg/m^2^ significantly decreased (*P*<0.05) from baseline to 90 days. In addition, the prevalence of HOMA-IR ≥3.0 significantly increased from baseline to 90 days (*P*<0.001).

Analysis of HDL-c after redefining low HDL-c as <1.04 mmol/L for both men and women still showed a significant decrease from baseline to 90 days (baseline: 72.9% vs. 90-day: 21.2%, *P*<0.001, respectively). Although there were nominal elevations in the prevalence of LDL-c ≥3.37 mmol/L (≥130 mg/dL) and glucose >5.55 mmol/L (>100 mg/dL), the changes were not statistically significant (*P*=0.48 and *P*=0.17, respectively). There was no change in the prevalence of triglycerides ≥1.70 mmol/L (≥150 mg/dL, *P*=0.83).

### Additional analyses

A comparison between participants whose data were included vs. excluded from the current analysis showed that the two groups were similar (*P*>0.05) with regard to sex, BMI, waist circumference, hip circumference, mid-upper arm circumference, smoking, CD4 cell count, CRP, serum albumin, TG, HDL-c, TC:HDL-c ratio, and intakes of alcohol, total energy and % energy from total fat, monounsaturated fat, polyunsaturated fat, saturated fat, proteins and carbohydrates. However, excluded participants had a significantly lower mean age compared to those included in the study (32.7±6.4 SD years vs. 35.0±7.9 years in those included, *P*=0.03). In addition, excluded participants had lower mean LDL-c (1.82±0.77 SD mmol/L vs. 2.12±0.75 mmol/L in those included, *P*=0.01) and TC (3.30±1.03 mmol/L vs. 3.56±0.83 mmol/L in those included, *P*=0.04).

## Discussion

This study showed that in the first 90 days, cART regimens prescribed to HIV/AIDS patients in Zambia in 2007 were associated with moderate increases in LDL-c and TC and substantial increases in HDL-c, leading to a substantial reduction in the TC:HDL-c ratio. Serum insulin, glucose and the HOMA-IR index also increased, suggesting a tendency towards insulin resistance. These changes were independent of the cART regimen.

The observed short-term lipid and metabolic changes are not likely to be associated with an increase in the risk of adverse cardiovascular outcomes since they mostly remained below established cut-points for increased cardiovascular disease risk in developed countries. The actual lipid cut-points associated with cardiovascular risk in developing countries are unknown. The significant increase in HDL-c and the consequent decrease in the TC:HDL-c ratio may suggest a cardioprotective effect of antiretroviral therapy [7,10,24,25]. This finding is consistent with results from the 2NN trial in which NVP compared to EFV conferred a better lipid profile [26]. The overall improved lipid profile in our study could in part be due to the fact that 95% of the patients were on NVP-based therapy. The observed improved lipid profile is of great interest given previous reports have suggested increased cardiovascular risk by HIV itself as well as by some cART [1,3]. The marginally significant difference in TC change among the three cART regimens was driven by the change in the ‘other’ group which, although smaller, was similar in direction to the change in the two main cART regimens. In addition to comprising many cART regimens (including AZT and D4T), the ‘other’ group has a small sample size (n=17), making the observed value more likely due to chance.

In agreement with our study findings, a two-year prospective study conducted in Uganda by Buchacz *et al.*[13] among 374 cART-naïve patients showed that TC and HDL-c were significantly higher (*P*<0.05) at one and two years following initiation of therapy compared to the baseline values. Buchacz *et al.* also showed that the TC:HDL-c ratio was significantly lower at one and two years compared to the baseline value. Additionally, a cross-sectional study among HIV/AIDS patients initiating cART in Tanzania reported lipid values that are comparable to those observed in the current study [14]. Furthermore, concordant with our findings, cross-sectional studies in Kenya [27] and India [28] comparing cART-naïve and treated groups showed that patients on cART had significantly higher (*P*<0.05) TC, LDL-c and HDL-c.

Moreover, in agreement with our findings, a study conducted in Cameroon by Yone *et al.*[29] compared cART-naïve HIV/AIDS patients with patients who had been on first-line cART for 12 months and reported that patients on cART had higher TC and LDL-c. Contrary to our findings, Yone *et al.* reported that patients on cART had similar HDL-c concentration with cART-naïve patients and also a higher TC:HDL-c ratio. Due to the cross-sectional study design, it is impossible to know whether the HDL-c concentration and the TC:HDL-c ratio among patients on cART improved or worsened following initiation of therapy. In addition, this study [29] only included patients who did not switch medications, which may have introduced selection bias.

Our study findings also indicate a significant increase (*P*<0.05) in serum insulin concentration, glucose and HOMA-IR index in the AZT+3TC+NVP and D4T+3TC+NVP cART regimen groups, suggesting that cART may increase insulin resistance [22,30]. Similar observations have been reported by Pujari *et al.* in India [28]. Moreover, studies from developed countries using the euglycemic clamp protocol to assess insulin resistance have shown that NRTIs, particularly AZT or D4T, are associated with increased insulin resistance [31,32]. Our results should be corroborated using more accurate measures of insulin resistance; if confirmed, it may be a concern because insulin resistance is an established risk factor for diabetes mellitus and cardiovascular disease [33,34].

About 44% of the recruited patients were excluded, mainly due to early termination of the study [17], raising a concern for selection bias. A comparison between patients whose data were included to those whose data were excluded indicates that the excluded patients were younger, had lower LDL-c and lower TC but similar in most other characteristics at baseline. Although age is a known modulator of cardiovascular risk [10], little is known about the effect of age on the risk for adverse cardiovascular outcomes in young people, and we speculate that a 2.3 years age difference between those included and those excluded in a young population may not result in a clinically meaningful risk difference. In addition, despite the slight differences in LDL-c and TC, the two groups had similar HDL-c and TC:HDL-c ratios, suggesting that they did not differ with regard to risk for adverse cardiovascular outcomes [10]. It is also noteworthy that despite the lipid differences, the mean TC level for the included and excluded patients was still much lower than the 200 mg/dL cut-point. Moreover, the two groups were similar in composition by sex, anthropometric measures, nutritional status, and serum inflammatory marker levels, all of which are strong modulators of cardiometabolic risk [10,35-40].

Unlike studies conducted in developed countries, none of the participants in our study reported use of any lipid-lowering drugs, eliminating confounding from such medications.

We acknowledge a number of limitations in our study. First, the study lacked a control group which makes it difficult to differentiate the effect of regression to the mean from the effect of cART. Second, we were not able to measure CRP concentrations at the 90-day follow-up. CRP is a strong correlate of cardiometabolic risk [3]. Third, we lacked baseline viral load values because they were not part of standard of care in Zambia at the time of the study and could not be measured for the current analysis due to financial constraints. Viral load measurements are important markers of disease progression in HIV infected patients and could influence response to antiretroviral therapy. However, the 3 cART regimen categories had similar CD4 cell counts (*P*=0.87) indicating no obvious differences in disease progression between the 3 treatment categories. Fourth, our study had a short follow-up period of 90 days, a period during which underweight or emaciated patients may still be resetting their metabolic profiles following treatment. However, the few studies in resource poor settings that have followed patients for a longer period (up to 24 months) have reported similar associations [13].

Nevertheless, larger and longer studies are warranted to examine the effects of different cART regimens on cardiometabolic risk and outcomes in sub-Saharan African populations.

## Conclusions

Our results suggest that first-line cART regimens in Zambia that did not include protease inhibitors are associated with cardioprotective lipid profiles characterized by a substantial increase in HDL-c and a decrease in the TC:HDL-c ratio. Our findings of increased insulin and HOMA-IR suggest a tendency towards insulin resistance. These results appear to be independent of the cART regimen used.

## Abbreviations

3TC: Lamivudine; AIDS: Acquired immune deficiency syndrome; AZT: Zidovudine; BMI: Body mass index; cART: Combination antiretroviral therapy; CRP: C-reactive protein; D4T: Stavudine; EFV: Efavirenz; FTC: Emtricitabine; HDL-c: High density lipoprotein cholesterol; HIV: Human immunodeficiency virus; LDL-c: Low density lipoprotein cholesterol; NVP: Nevirapine; TC: Total cholesterol; TDF: Tenofovir; TG: Triglycerides.

## Competing interests

The authors declare that they have no competing interests.

## Authors’ contributions

Study conception and design: EKK, DCH and DKA. Participant recruitment: EKK, DCH, CKN, SB and BHC. Manuscript draft: JNK. Data analysis, interpretation and critical review of the manuscript: JNK, DCH, CKN, MFW, SB, JRK, BHC, DKA and EKK. All authors read and approved the final manuscript.

## References

[B1] Friis-Moller N Sabin CA Weber R D’Arminio Monforte A El-Sadr WM Reiss P Thiebaut R Morfeldt L De Wit S Pradier C Combination antiretroviral therapy and the risk of myocardial infarction N Engl J Med 2003 349 1993 2003 14627784 10.1056/NEJMoa030218

[B2] Triant VA Lee H Hadigan C Grinspoon SK Increased acute myocardial infarction rates and cardiovascular risk factors among patients with human immunodeficiency virus disease J Clin Endocrinol Metab 2007 92 2506 2512 10.1210/jc.2006-2190 17456578 PMC2763385

[B3] Brown TT Cole SR Li X Kingsley LA Palella FJ Riddler SA Visscher BR Margolick JB Dobs AS Antiretroviral therapy and the prevalence and incidence of diabetes mellitus in the multicenter AIDS cohort study Arch Intern Med 2005 165 1179 1184 10.1001/archinte.165.10.1179 15911733

[B4] Dube MP Stein JH Aberg JA Fichtenbaum CJ Gerber JG Tashima KT Henry WK Currier JS Sprecher D Glesby MJ Guidelines for the evaluation and management of dyslipidemia in human immunodeficiency virus (HIV)-infected adults receiving antiretroviral therapy: recommendations of the HIV Medical Association of the Infectious Disease Society of America and the Adult AIDS Clinical Trials Group Clin Infect Dis 2003 37 613 627 10.1086/378131 12942391

[B5] Jones R Sawleshwarkar S Michailidis C Jackson A Mandalia S Stebbing J Bower M Nelson M Gazzard BG Moyle GJ Impact of antiretroviral choice on hypercholesterolaemia events: the role of the nucleoside reverse transcriptase inhibitor backbone HIV Med 2005 6 396 402 10.1111/j.1468-1293.2005.00325.x 16268821

[B6] van der Valk M Kastelein JJ Murphy RL van Leth F Katlama C Horban A Glesby M Behrens G Clotet B Stellato RK Nevirapine-containing antiretroviral therapy in HIV-1 infected patients results in an anti-atherogenic lipid profile AIDS 2001 15 2407 2414 10.1097/00002030-200112070-00008 11740191

[B7] Fontas E van Leth F Sabin CA Friis-Moller N Rickenbach M D’Arminio Monforte A Kirk O Dupon M Morfeldt L Mateu S Lipid profiles in HIV-infected patients receiving combination antiretroviral therapy: are different antiretroviral drugs associated with different lipid profiles? J Infect Dis 2004 189 1056 1074 10.1086/381783 14999610

[B8] Passalaris JD Sepkowitz KA Glesby MJ Coronary artery disease and human immunodeficiency virus infection Clin Infect Dis 2000 31 787 797 10.1086/313995 11017831

[B9] Iloeje UH Yuan Y L’Italien G Mauskopf J Holmberg SD Moorman AC Wood KC Moore RD Protease inhibitor exposure and increased risk of cardiovascular disease in HIV-infected patients HIV Med 2005 6 37 44 10.1111/j.1468-1293.2005.00265.x 15670251

[B10] National Cholesterol Education Program Expert Panel on Detection EaToHBCiA Third report of the national cholesterol education program (NCEP) expert panel on detection, evaluation, and treatment of high blood cholesterol in adults (Adult Treatment Panel III) final report Circulation 2002 106 3143 3421 12485966

[B11] Group DADS Friis-Moller N Reiss P Sabin CA Weber R Monforte A El-Sadr W Thiebaut R De Wit S Kirk O Class of antiretroviral drugs and the risk of myocardial infarction N Engl J Med 2007 356 1723 1735 17460226 10.1056/NEJMoa062744

[B12] Group DADS Sabin CA Worm SW Weber R Reiss P El-Sadr W Dabis F De Wit S Law M D’Arminio Monforte A Use of nucleoside reverse transcriptase inhibitors and risk of myocardial infarction in HIV-infected patients enrolled in the D:A:D study: a multi-cohort collaboration Lancet 2008 371 1417 1426 18387667 10.1016/S0140-6736(08)60423-7 PMC2688660

[B13] Buchacz K Weidle PJ Moore D Were W Mermin J Downing R Kigozi A Borkowf CB Ndazima V Brooks JT Changes in lipid profile over 24 months among adults on first-line highly active antiretroviral therapy in the home-based AIDS care program in rural Uganda J Acquir Immune Defic Syndr 2008 47 304 311 10.1097/QAI.0b013e31815e7453 18398971

[B14] Armstrong C Liu E Okuma J Spiegelman D Guerino C Njelekela M Grinspoon S Fawzi W Hawkins C Dyslipidemia in an HIV-positive antiretroviral treatment-naive population in Dar es Salaam, Tanzania J Acquir Immune Defic Syndr 2011 57 141 145 10.1097/QAI.0b013e318219a3d1 21436713 PMC3125454

[B15] Gupta A Nadkarni G Yang WT Chandrasekhar A Gupte N Bisson GP Hosseinipour M Gummadi N Early mortality in adults initiating antiretroviral therapy (ART) in low- and middle-income countries (LMIC): a systematic review and meta-analysis PLoS One 2011 6 e28691 10.1371/journal.pone.0028691 22220193 PMC3248405

[B16] Egger M Boulle A Schechter M Miotti P Antiretroviral therapy in resource-poor settings: scaling up inequalities? Int J Epidemiol 2005 34 509 512 England 15941732 10.1093/ije/dyi110

[B17] Ngu JN Heimburger DC Arnett DK Nyirenda CK Potter D Zulu I Bosire CN Bagchi S Ye J Chi BH Kabagambe EK Fasting triglyceride concentrations are associated with early mortality following antiretroviral therapy in Zambia N A J Med Sci 2010 3 79 88 10.7156/v3i2p079 PMC3207243 22059107

[B18] Heimburger DC Koethe JR Nyirenda C Bosire C Chiasera JM Blevins M Munoz AJ Shepherd BE Potter D Zulu I Serum phosphate predicts early mortality in adults starting antiretroviral therapy in Lusaka, Zambia: a prospective cohort study PLoS One 2010 5 e10687 10.1371/journal.pone.0010687 20502700 PMC2872675

[B19] Chi BH Mwango A Giganti M Mulenga LB Tambatamba-Chapula B Reid SE Bolton-Moore C Chintu N Mulenga PL Stringer EM Early clinical and programmatic outcomes with tenofovir-based antiretroviral therapy in Zambia J Acquir Immune Defic Syndr 2010 54 63 70 20009765 10.1097/QAI.0b013e3181c6c65c PMC2862003

[B20] American Diabetes A Diagnosis and classification of diabetes mellitus Diabetes Care 2008 31 Suppl 1 S55 S60 18165338 10.2337/dc08-S055

[B21] Cakir MSR Tosun O Saka O Karayalcin U Reproducubility of fasting and OGTT-derived insulin resistance indices in normoglycemic women Can J Diabetes 2006 30 46 51

[B22] Lee S Choi S Kim HJ Chung YS Lee KW Lee HC Huh KB Kim DJ Cutoff values of surrogate measures of insulin resistance for metabolic syndrome in Korean non-diabetic adults J Korean Med Sci 2006 21 695 700 10.3346/jkms.2006.21.4.695 16891815 PMC2729893

[B23] Matthews DR Hosker JP Rudenski AS Naylor BA Treacher DF Turner RC Homeostasis model assessment: insulin resistance and beta-cell function from fasting plasma glucose and insulin concentrations in man Diabetologia 1985 28 412 419 10.1007/BF00280883 3899825

[B24] Grunfeld C Dyslipidemia and its treatment in HIV infection Top HIV Med 2010 18 112 118 20921577 PMC3189481

[B25] Duprez DA Kuller LH Tracy R Otvos J Cooper DA Hoy J Neuhaus J Paton NI Friis-Moller N Lampe F Lipoprotein particle subclasses, cardiovascular disease and HIV infection Atherosclerosis 2009 207 524 529 10.1016/j.atherosclerosis.2009.05.001 19515371 PMC2818719

[B26] van Leth F Phanuphak P Stroes E Gazzard B Cahn P Raffi F Wood R Bloch M Katlama C Kastelein JJ Nevirapine and efavirenz elicit different changes in lipid profiles in antiretroviral-therapy-naive patients infected with HIV-1 PLoS Med 2004 1 e19 10.1371/journal.pmed.0010019 15526045 PMC523838

[B27] Manuthu EM Joshi MD Lule GN Karari E Prevalence of dyslipidemia and dysglycaemia in HIV infected patients East Afr Med J 2008 85 10 17 18543521 10.4314/eamj.v85i1.9600

[B28] Pujari SN Dravid A Naik E Bhagat S Tash K Nadler JP Sinnott JT Lipodystrophy and dyslipidemia among patients taking first-line, World Health Organization-recommended highly active antiretroviral therapy regimens in Western India J Acquir Immune Defic Syndr 2005 39 199 202 15905737

[B29] Pefura Yone EW Betyoumin AF Kengne AP Kaze Folefack FJ Ngogang J First-line antiretroviral therapy and dyslipidemia in people living with HIV-1 in Cameroon: a cross-sectional study AIDS Res Ther 2011 8 33 10.1186/1742-6405-8-33 21943115 PMC3197472

[B30] Bonora E Targher G Alberiche M Bonadonna RC Saggiani F Zenere MB Monauni T Muggeo M Homeostasis model assessment closely mirrors the glucose clamp technique in the assessment of insulin sensitivity: studies in subjects with various degrees of glucose tolerance and insulin sensitivity Diabetes Care 2000 23 57 63 10.2337/diacare.23.1.57 10857969

[B31] van Vonderen MG Blumer RM Hassink EA Sutinen J Ackermans MT van Agtmael MA Yki-Jarvinen H Danner SA Serlie MJ Sauerwein HP Reiss P Insulin sensitivity in multiple pathways is differently affected during zidovudine/lamivudine-containing compared with NRTI-sparing combination antiretroviral therapy J Acquir Immune Defic Syndr 2010 53 186 193 10.1097/QAI.0b013e3181c190f4 19898246

[B32] Fleischman A Johnsen S Systrom DM Hrovat M Farrar CT Frontera W Fitch K Thomas BJ Torriani M Cote HC Grinspoon SK Effects of a nucleoside reverse transcriptase inhibitor, stavudine, on glucose disposal and mitochondrial function in muscle of healthy adults Am J Physiol Endocrinol Metab 2007 292 E1666 E1673 10.1152/ajpendo.00550.2006 17284576 PMC3206591

[B33] Groop LC Insulin resistance: the fundamental trigger of type 2 diabetes Diabetes Obes Metab 1999 1 Suppl 1 S1 S7 11220283 10.1046/j.1463-1326.1999.0010s1001.x

[B34] Cavaghan MK Ehrmann DA Polonsky KS Interactions between insulin resistance and insulin secretion in the development of glucose intolerance J Clin Invest 2000 106 329 333 10.1172/JCI10761 10930434 PMC314336

[B35] Poirier P Giles TD Bray GA Hong Y Stern JS Pi-Sunyer FX Eckel RH American Heart A Obesity and cardiovascular disease: pathophysiology, evaluation, and effect of weight loss: an update of the 1997 American heart association scientific statement on obesity and heart disease from the Obesity Committee of the Council on nutrition, physical activity, and metabolism Circulation 2006 113 898 918 10.1161/CIRCULATIONAHA.106.171016 16380542

[B36] Liu M Chan CP Yan BP Zhang Q Lam YY Li RJ Sanderson JE Coats AJ Sun JP Yip GW Yu CM Albumin levels predict survival in patients with heart failure and preserved ejection fraction Eur J Heart Fail 2012 14 39 44 10.1093/eurjhf/hfr154 22158777

[B37] Kabagambe EK Judd SE Howard VJ Zakai NA Jenny NS Hsieh M Warnock DG Cushman M Inflammation biomarkers and risk of all-cause mortality in the reasons for geographic and racial differences in stroke cohort Am J Epidemiol 2011 174 284 292 10.1093/aje/kwr085 21685411 PMC3202158

[B38] Danesh J Wheeler JG Hirschfield GM Eda S Eiriksdottir G Rumley A Lowe GD Pepys MB Gudnason V C-reactive protein and other circulating markers of inflammation in the prediction of coronary heart disease N Engl J Med 2004 350 1387 1397 10.1056/NEJMoa032804 15070788

[B39] Ridker PM Hennekens CH Buring JE Rifai N C-reactive protein and other markers of inflammation in the prediction of cardiovascular disease in women N Engl J Med 2000 342 836 843 10.1056/NEJM200003233421202 10733371

[B40] Mak KH Bhatt DL Shao M Haffner SM Hamm CW Hankey GJ Johnston SC Montalescot G Steg PG Steinhubl SR The influence of body mass index on mortality and bleeding among patients with or at high-risk of atherothrombotic disease Eur Heart J 2009 30 857 865 19233855 10.1093/eurheartj/ehp037

